# Non-pharmacological interventions designed to reduce health risks due to unhealthy eating behaviour and linked risky or excessive drinking in adults aged 18–25 years: a systematic review protocol

**DOI:** 10.1186/s13643-017-0434-6

**Published:** 2017-03-03

**Authors:** Stephanie Scott, Kathryn Parkinson, Eileen Kaner, Shannon Robalino, Martine Stead, Christine Power, Niamh Fitzgerald, Wendy Wrieden, Ashley Adamson

**Affiliations:** 1 Institute of Health & Society, Baddiley-Clark Building, Newcastle University, Richardson Road, Newcastle upon Tyne, NE2 4AX UK; 2 Institute for Social Marketing (ISM), University of Stirling, Stirling, FK9 4LA UK; 3 Population, Policy and Practice, UCL Great Ormond Street Institute of Child Health, 30 Guilford Street, London, WC1N 1EH UK

**Keywords:** Intervention, Young adult, Alcohol, Eating behaviour, Systematic review

## Abstract

**Background:**

Excess body weight and heavy alcohol consumption are two of the greatest contributors to global disease. Alcohol use peaks in early adulthood. Alcohol consumption can also exacerbate weight gain. A high body mass index and heavy drinking are independently associated with liver disease but, in combination, they produce an intensified risk of damage, with individuals from lower socio-economic status groups disproportionately affected.

**Methods:**

We will conduct searches in MEDLINE, Embase, PubMed, PsycINFO, ERIC, ASSIA, Web of Knowledge (WoK), Scopus, CINAHL via EBSCO, LILACS, CENTRAL and ProQuest Dissertations and Theses for studies that assess targeted preventative interventions of any length of time or duration of follow-up that are focused on reducing unhealthy eating behaviour and linked risky alcohol use in 18–25-year-olds. Primary outcomes will be reported changes in: (1) dietary, nutritional or energy intake and (2) alcohol consumption. We will include all randomised controlled trials (RCTs) including cluster RCTs; randomised trials; non-randomised controlled trials; interrupted time series; quasi-experimental; cohort involving concurrent or historical controls and controlled before and after studies. Database searches will be supplemented with searches of Google Scholar, hand searches of key journals and backward and forward citation searches of reference lists of identified papers. Search records will be independently screened by two researchers, with full-text copies of potentially relevant papers retrieved for in-depth review against the inclusion criteria. Methodological quality of RCTs will be evaluated using the Cochrane risk of bias tool. Other study designs will be evaluated using the Cochrane Public Health Review Group’s recommended Effective Public Health Practice Project Quality Assessment Tool for Quantitative Studies. Studies will be pooled by meta-analysis and/or narrative synthesis as appropriate for the nature of the data retrieved.

**Discussion:**

It is anticipated that exploration of intervention effectiveness and characteristics (including theory base, behaviour change technique; modality, delivery agent(s) and training of intervention deliverers, including their professional status; and frequency/duration of exposure) will aid subsequent co-design and piloting of a future intervention to help reduce health risk and social inequalities due to excess weight gain and alcohol consumption.

**Systematic review registration:**

PROSPERO CRD42016040128.

**Electronic supplementary material:**

The online version of this article (doi:10.1186/s13643-017-0434-6) contains supplementary material, which is available to authorized users.

## Background

Excess body weight and heavy alcohol consumption are two of the greatest contributors to global disease burden [1, 2]. Being overweight and/or obese accounts for 5% of deaths worldwide [2] and is responsible for raising the risk of chronic diseases such as diabetes, heart diseases and cancers. Heavy alcohol consumption contributes to over 200 disease and injury conditions and is responsible for almost 6% of world deaths [1]. Risky or heavy alcohol use is the leading risk to health and wellbeing in young people, accounting for 7% of disability adjusted life years in 10–24-year-olds globally [3]. Rates of liver disease are linked to both alcohol use and obesity and they are rising rapidly in the UK [4, 5], particularly in those aged below 44 years [4]. Over the past 30 years, the UK has seen a fourfold increase in liver disease mortality and it is now the third most common cause of premature death, with 62,000 years of working life lost each year [6]. Most of these deaths are alcohol-related [7]. However, Non-alcohol fatty liver disease (NAFLD) is becoming increasingly common and it is now the most prevalent liver disorder in children and young adults with overall prevalence of between 2.6 and 9.8% in overweight individuals which rises to between 42 and 77% in those who are obese [6]. In addition, it has been shown that the combination of a raised body mass index (BMI) and heavy alcohol consumption can result in an intensified interaction creating a steeply elevated risk of liver disease in men and women [8] and that heavy drinking is associated with greater waist-hip-ratio in mid-life even when taking other lifetime influences into account [9].

Research on reducing excess body weight or heavy alcohol consumption typically occurs in isolation or as part of non-specific multiple behaviour change interventions. Recent exceptions have been behavioural modification work with middle-aged obese men who were also alcohol drinkers [10, 11] and the BeWEL study which sought to reduce weight by addressing diet, physical activity and alcohol amongst overweight or obese middle-aged adults at increased risk of colorectal cancer and other obesity-related comorbidities [12]. In the latter study, attention paid to alcohol was less than that paid to diet and physical activity. Strategies to jointly reduce alcohol consumption and address levels of overweight or obesity may produce greater health gains and be a more efficient use of resources than initiatives directed towards each pattern alone. As such, understanding this relationship is a global public health priority [8], particularly amongst younger people [13]. Adverse health behaviours begin to cluster during adolescence [14], and excess body weight and risky drinking have both been demonstrated to track into and throughout adulthood [15–17]. Consequently, there is a strong rationale to intervene with young adults early; before these behaviour patterns become fully entrenched habits which lead to poorer health and social outcomes later in life. Further, there is increasing interest in developing and evaluating integrative interventions targeting multiple risk behaviours [18–20].

Many eating rituals have become strongly linked to the use of alcohol and vice versa; for instance, salty snacks are often sold in public drinking venues and there is a popular concept of drinking alcohol with dinner or visiting fast food outlets after an evening out at drinking establishments [21]. Indeed, it has been suggested that unhealthy food choices are more likely to be made during and directly after a period of prolonged alcohol consumption [21] which could be due, at least in part, to the disinhibiting effect of alcohol which is a psychoactive substance that can alter usual behaviour. However, there are key differences when thinking about eating and unhealthy drinking behaviour. All individuals need to eat to survive, whilst many individuals choose to drink alcohol because it is perceived to be a pleasurable component of social life. Alcohol contains energy, but it is a nutritionally poor food source and does not stimulate satiety [22]. This may make it more likely for alcohol calories to be consumed in addition to energy intake from food.

Epidemiological data suggest that energy intake from alcohol, type of beverage and drinking pattern (i.e. high volume, high frequency) is associated with excess body weight and weight gain amongst adults [22, 23]. Few studies have explored this relationship amongst young adults. Those that do have shown a positive association between being overweight and/or obese and alcohol consumption, particularly amongst females [24, 25], whilst others have highlighted a conflict for some individuals between a wish to stay slim and also to drink alcohol as part of developing a social identity [26, 27]. Furthermore, there have been some reports of individuals choosing not to eat prior to socialising, so that they can drink alcohol and avoid weight gain; a phenomenon that has been termed ‘Drunkorexia’ [26]. This increases the likelihood of intoxication, where blood alcohol levels rise sharply and affect the brain and subsequent behaviour, which steeply increases the risk of acute harm from drinking.

Recent qualitative research demonstrates that young adults make trade-offs between food and alcohol consumption and levels of physical activity undertaken [28]. Little more is known about young adult’s perspectives on the relationship between alcohol consumption and unhealthy eating behaviour, i.e. patterns of food choice or behaviours that lead to adverse health outcomes, such as snacking, eating energy rich or high-sugar foods or avoiding eating so that alcohol calories do not lead to weight gain. It is increasingly recognised that health promoting interventions must acknowledge social and emotional needs [29, 30] as well as focus on reducing health risks. Recent work has begun to investigate wider cultural drivers of drinking and eating behaviour in early adolescence [31, 32], but this needs to extend to early adulthood in order to understand lifestyle choices in terms of co-occurring behaviours and wider socio-cultural drivers, and informs subsequent interventions.

Whilst a current Cochrane systematic review examines individual-, family- and school-led interventions for preventing multiple risk behaviours in individuals aged 8 to 25 years [19], no systematic review has examined the specific impact of interventions to reduce unhealthy eating and risky drinking and their health consequences amongst adults or individuals aged 18–25 years only. The systematic review proposed here will address this evidence gap by reviewing primary studies that have considered the effectiveness of preventative interventions focused on reducing unhealthy eating behaviour and linked risky alcohol use in young adults aged 18–25. We will focus on targeted interventions to improve health behaviour at an early stage of risk or harm, when it is likely to be most amenable to change. Such measures tend to include screening to identify relevant individuals, followed by the delivery of individual feedback, advice and/or counselling [33, 34].

## Objectives

The primary objective of this review is to systematically evaluate the current evidence-base and determine the effectiveness of preventative targeted interventions focused on reducing unhealthy eating behaviour and linked risky alcohol use and their health consequences in adults aged 18–25 years.

## Methods

### Study registration

The review will be carried out following established criteria for the good conduct and reporting of systematic reviews [35]. The protocol was structured according to the Preferred Reporting Items of Systematic Reviews and Meta-Analyses Protocol (PRISMA-P) guidelines [36] (see Additional file 1) and is registered with the PROSPERO International Prospective Register of Systematic Reviews (Ref: CRD42016040128).

### Inclusion criteria

#### Types of studies

We will include the following study designs: randomised controlled trials (including cluster RCTs); randomised trials; non-randomised controlled trials (e.g. studies with multiple clusters/communities where allocation to interventions was not randomised); interrupted time series; quasi-experimental; cohort involving concurrent or historical controls and controlled before and after studies. Unpublished data, abstracts and conference proceedings will not be included. Studies including no primary evaluation data (e.g. protocols, editorials, reviews) will also be excluded, as well as studies presenting qualitative data only. For studies using mixed methods, only data relating to the quantitative evaluation will be considered. Studies will be considered where both behaviours are addressed simultaneously (multicomponent interventions) or where one behaviour is the focus of the intervention content but the other is the context of its delivery (e.g. alcohol intervention in overweight or obese individuals).

#### Types of participants

This review will focus on interventions targeted at free-living (not mandated, hospitalised or imprisoned) male and female young adults aged 18–25 years screened for unhealthy eating and risky drinking behaviours in any country, and whose outcomes are assessed for this group. Individuals without dietary risks or who do not currently consume alcohol excessively will be excluded. Studies may subsequently be stratified to account for inter-country differences in overweight/obesity and risky drinking. The review will also include interventions targeted at wider population groups where outcomes have been assessed for this particular subgroup. Studies will be excluded where the study population: (a) requires specialist treatment for alcohol dependency or weight loss and gain (i.e. bariatric surgery) and (b) pregnant or breastfeeding women whose current eating pattern may be time-limited and not reflective of usual diet behaviours.

#### Types of intervention(s)

This review will consider studies which evaluate behaviour modification strategies (at the individual, community and societal level) based on information, advice and counselling targeting unhealthy eating and linked risky alcohol use. These interventions may be delivered in person, by telephone, by internet or a combination of multiple delivery methods. The interventions may be delivered individually, as part of a group or a combination in any setting. Pharmacological and laboratory-based interventions will be excluded. There will be no restrictions in terms of length of intervention or follow-up.

#### Types of comparators

Interventions will be compared to control (no intervention or waiting list), screening/assessment only and treatment as usual.

#### Types of outcomes

Primary outcomes variables will be reported changes in: (1) dietary, nutritional or energy intake and (2) alcohol consumption. Examples of accepted measures will include, but are not restricted to, self-report, researcher observations, relevant validated questionnaires or indicators such as food-frequency, fruit and vegetable intake, delayed first drink, reduction in total volume consumption, Alcohol Use Disorders Identification Test (AUDIT) and Time Line Follow Back (TLFB), photographs of food portions and weighed intake. Secondary outcome variables will be measured of body composition and alcohol-related outcomes which do not focus directly on consumption. Examples of accepted measures will include BMI, waist circumference, waist-hip ratio, % body fat, purchasing behaviour and hospital admissions. We will extract outcomes in all data forms (e.g. dichotomous, continuous) as reported in the included studies. Guided by existing work on behaviour change techniques [37, 38], theory, modality of intervention and delivery agent, as well as measures of attrition/retention rates, will also be captured.

### Search strategy

A three-step search strategy will be undertaken. A scoping search of MEDLINE and CINAHL will be used to identify keywords and phrases in the paper title and abstract and MeSH/thesaurus terms used to index relevant articles. A second search using identified keywords and thesaurus terms will subsequently be undertaken across all included databases. Thesaurus terms will be translated as appropriate across databases. Trial registers (World Health Organization International Clinical Trials Registry; Meta-Register of Controlled Trials) will also be searched for relevant studies that may have resulted in publications not identified using the electronic database search. Finally, database searches will be supplemented with general searches of the internet, searches of Google Scholar for relevant studies based on key names of field experts; grey literature searches; and backward-and-forward hand searches of reference lists of included papers and relevant reviews. We will not exclude papers on the basis of language, country or publication date. The following databases will be searched: MEDLINE, Embase, PubMed, PsycINFO, ERIC, ASSIA, Web of Knowledge, Scopus, CINAHL, LILACS, CENTRAL and ProQuest Dissertations and Theses. A provisional full search strategy (to be confirmed following scoping searches) is presented in Table 1.

**Table 1:** Sample search strategy

1. (multi* adj2 health* adj2 behavio?r*).ti,ab.
2. (multi* adj2 unhealth* adj2 behavio?r*).ti,ab.
3. (multi* adj2 risk* adj2 behavio?r*).ti,ab.
4. (co?variat* adj5 behavio?r*).ti,ab.
5. (cluster* adj2 health* adj2 behavio?r*).ti,ab.
6. (cluster* adj2 unhealth* adj2 behavio?r*).ti,ab.
7. (cluster* adj2 risk* adj2 behavio?r*).ti,ab.
8. (risk-taking adj2 behavio?r*).ti,ab.
9. risk-taking/
10. or/1-9
11. exp Alcohol Drinking/
12. exp alcoholic beverages/
13. alcoholic intoxication/
14. (alcohol* adj2 (abuse* or misuse* or use* or consum* or drink* or excess* or problem* or risk*)).mp.
15. alcohol*.mp.
16. ((binge or problem* or risk* or excess*) adj2 drink*).mp.
17. ((hazardous or unsafe or unhealthy) adj2 drink*).mp.
18. drunk*.mp.
19. (intoxicat* adj4 (drink* or alcohol*)).mp.
20. (wine or beer or spirits).mp.
21. or/11-20
22. exp overweight/
23. exp overnutrition/or hyperphagia/
24. exp “Body Weights and Measures”/
25. Food preferences/
26. *Feeding Behavior/or exp Food habits/
27. Energy Intake/
28. fast foods/or carbonated beverages/
29. (obes* or over?weight or over?nutrition).ti,ab.
30. (excess adj2 weight).ti,ab.
31. ((eat* or food* or feed*) adj2 (behavio?r* or excessive* or choice? or pattern? or habit? or preference?)).ti,ab.
32. (body adj2 (mass or size or weight)).ti,ab.
33. (diet* or nutrition).ti,ab.
34. over?eat*.mp.
35. (under?weight or under?nutrition or under?eat).ti,ab.
36. (unhealth* adj2 (diet* or eating or food*)).mp.
37. ((vegetable* or fruit) adj2 (eat* or intake or consum* or portion* or serving? or frequenc* or number? or preference? or choice*)).mp.
38. (((junk or fast or unhealthy or choice? or processed) adj2 food*) or fastfood).mp.
39. (calorie-dense adj2 (food? or beverage? or drink?)).mp.
40. (convenien* adj2 (food* or meal*)).mp.
41. (excess* adj2 (fat* or salt* or sugar*)).mp.
42. (energy adj1 intake).mp.
43. (poor adj2 diet).mp.
44. snack*.mp.
45. (((fizzy or sugary) adj2 drink*) or soda or coca-cola or coke or cola or pop).mp.
46. (take?away or take?out or carry?out).mp.
47. (((frozen or ready or TV or television) adj2 meal?) or ((TV or television) adj2 dinner?)).mp.
48. ((portion or serving) adj2 size?).mp.
49. or/22-48
50. Young Adult/
51. (young adj2 (adult? or person?)).mp.
52. ((college* or university) adj2 student?).mp.
53. late-teen*.mp.
54. early-adult*.mp.
55. (adolescen* or youth* or undergraduate* or freshmen or fresher? or teen* or student?).mp.
56. or/50-55
57. intervention?.mp.
58. (weightloss or (weight adj2 (reduc* or loss))).mp.
59. ((decreas* or reduc*) adj2 (alcohol* or drink*)).mp.
60. ((improve* or health*) adj2 (diet* or food? or choice?)).mp.
61. Health Promotion/
62. Health behavior/
63. Primary prevention/
64. Secondary prevention/
65. *risk reduction behavior/
66. (health adj1 (promot* or protect*)).mp.
67. ((modif* or chang*) adj5 (behavio?r* or habit*)).mp.
68. (early adj2 therap*).mp.
69. prevent*.mp.
70. (person* adj2 feedback).mp.
71. or/57-70
72. ((10 and 21) or (10 and 49) or (21 and 49)) and 56 and 71
73. adult/not (adult/and (young adult/or adolescent/))
74. 72 not 73

*represents the PubMed symbol for truncation

### Data extraction

The title and abstract of all records retrieved will be downloaded to Endnote X7 and independently screened by two researchers (SS, KP), with full-text copies of potentially relevant papers retrieved for in-depth review against the inclusion criteria. Any uncertainties will be resolved by discussion and referral to a third party if necessary (EK). Reasons for exclusion will be noted at the full-text stage. A flow chart of the selection process, following PRISMA guidelines, will be produced. Independent dual data extraction will be carried out using a prepiloted form in an Excel spreadsheet. Study characteristics will include: country of origin; year of study and duration; study design and risk of bias assessment; participants’ characteristics (age, gender, ethnicity, socio-economic status, baseline BMI and alcohol use) and intervention characteristics (including theory base, behaviour change technique; modality, delivery agent(s) and training of intervention deliverers, including their professional status; and frequency/duration of exposure). Outcomes will focus on diet, nutritional or energy intake, BMI and other measures of body composition, alcohol use and alcohol-related problems.

### Data synthesis

Where possible, quantitative data will be pooled in statistical meta-analysis using RevMan 5.1. In order to synthesise data across studies, we will compute and report mean differences where identical scales are used to measure the same outcome. Where scales vary, we will compute standardised mean differences (SMDs) (otherwise weighted mean differences) between comparison groups. For dichotomous data, we will report odds ratios (ORs) with 95% confidence intervals (CIs). For continuous data, we will most likely report standardised mean differences and 95% confidence intervals. Statistical analysis of the data will be decided by assessing the quality of included studies and the degree of heterogeneity found.

### Assessing heterogeneity

We will assess heterogeneity by calculating *χ* ^2^ and *I* ^2^ values for all outcome variables. Statistical heterogeneity will be considered substantial if *I* ^2^ values are above 50%. We will also assess the impact of heterogeneity through sensitivity analyses and assume the appropriate random effects or fixed effect model in meta-analyses accordingly [39]. Thus, if minimal heterogeneity is observed, a random effect analysis will be performed for a more realistic estimate of effect size. If there is significant diversity in intervention components, outcomes assessed, measurement tools and time points, computing and pooling effect sizes are unlikely to be meaningful. Thus, where significant heterogeneity is present, a narrative synthesis will be carried out including tables and figures to aid data presentation and interpretation where appropriate. If sufficient data are available, we will perform a sensitivity analysis that only includes RCTs as the highest grade of evidence. Sensitivity analysis will also explore the effect of excluding studies that appear to be statistical outliers.

### Unit of analysis issues

We anticipate unit of analysis issues such as repeated observations of the same outcome, studies including multiple intervention arms, groups of individuals randomised together to the same intervention (cluster-randomised trials) and individuals undergoing more than one intervention (for example, in a crossover trial). If study authors have not taken into account of these issues, an attempt will be made to investigate subsequent risk of bias.

### Missing data

Where possible, authors will be contacted to ask for further information where the reporting of these was insufficient. This includes missing data on the methods used, intervention content or material, outcome or precision measures. If missing data required for analyses cannot be obtained from the study author or extrapolated from other statistics, the study will be excluded. Finally, we will address the potential impact of missing data on the review findings in the ‘Discussion’ section of subsequent publications and reports.

### Subgroup analysis

If sufficient data are available, a subgroup analysis will be conducted. Proposed subgroups include gender (men versus women), socio-economic status, ethnicity, setting (workplace versus college/university), and mode of intervention delivery.

### Assessment of methodological quality

Included studies will be assessed independently by two researchers (SS, KP) for their methodological quality. In order to minimise any selection bias, both reviewers will be blind to the source and the authorship of the paper. If the two reviewers disagree with the rating of any study, a third review author (EK) will be available to consult in order to reach a consensus. The Cochrane risk of bias tool will be used for any RCTs which are included in the analysis to estimate selection bias (differences between groups compared), performance bias (arising from participants being aware of the group they are randomised to), attrition bias (arising from subjects withdrawing from the trial) and detection bias (which refers to problems with outcome assessments) [39]. For each item, studies will be classified as ‘high’, ‘low’ or ‘unclear’ risk of bias. The unclear category will be used for papers for which there is insufficient detail for a conclusion to be reached. Publication bias will also be examined using funnel plots. To assess the quality of other types of studies, a tool adapted from the Cochrane Public Health Review Group’s recommended Effective Public Health Practice Project Quality Assessment Tool for Quantitative Studies will be used [40].

## Discussion

The review is situated within a broader mixed method research project. The results of this review will be used to inform in-depth qualitative work to explore the links between unhealthy eating behaviour and risky alcohol use in the social, emotional and cultural lives of young adults (aged 18–25), including perceptions of risks, benefits, costs and consequences of these behaviours in early adulthood. It is anticipated that exploration of intervention effectiveness and characteristics (including theory base, behaviour change technique; modality, delivery agent(s) and training of intervention deliverers, including their professional status; and frequency/duration of exposure) will aid subsequent co-design and piloting of a future intervention to help reduce health risk and social inequalities due to excess weight gain and alcohol consumption.

## Additional file

Additional file 1: PRISMA-P guidelines. (DOC 82 kb)

## Abbreviations

ASSIA: Applied Social Sciences Index and Abstracts
AUDIT: Alcohol Use Disorders Identification Test
BMI: Body mass index
CENTRAL: Cochrane Central Register of Controlled Trials
CI: Confidence interval
CINAHL: Cumulative Index to Nursing and Allied Health Literature
ERIC: Education Resources Information Center
LILACS: Latin American and Caribbean Health Sciences Literature
NAFLD: Non-alcohol fatty liver disease
NHS: National Health Service
NICE: National Institute for Health and Care Excellence
PRISMA-P: Preferred Reporting Items of Systematic Reviews and Meta-Analyses Protocol
RCT: Randomised controlled trial
SMD: Standardised mean differences
TLFB: Time Line Follow Back
UK: United Kingdom
WoK: Web of Knowledge
